# Cost and affordability of a healthy diet for urban populations in Thailand and the Philippines before and during the COVID-19 pandemic

**DOI:** 10.1186/s12889-023-16207-4

**Published:** 2023-07-20

**Authors:** Mercy Mwambi, Pepijn Schreinemachers, Suwanna Praneetvatakul, Jody Harris

**Affiliations:** 1 World Vegetable Center, P.O. Box 1010, Bangkok, 10903 Thailand; 2 Department of Agricultural and Resource Economics, Faculty of Economics, Kasetsart University, Bangkok, 10900 Thailand

**Keywords:** Cost of recommended diet, Food prices, Nutrition, Urban poor, Food security

## Abstract

**Background:**

The coronavirus disease 2019 (COVID-19) pandemic severely affected global food security, but analyses of its impact on the cost and affordability of a healthy diet are limited. This study examines the immediate effect of the COVID-19 pandemic on the cost and affordability of a healthy diet among urban households in Bangkok, Thailand and Manila, the Philippines.

**Methods:**

We used official food price and household income and food expenditure data from the national statistics offices. The cost of recommended diet (CoRD) method was employed to assess the minimum cost of a healthy diet, following the healthy diet recommendations provided in the national food-based dietary guidelines of the specific countries. Regression discontinuity design was estimated to determine the COVID-19 effect on food prices and scenario analysis done to determine the effect of reduced food budgets with and without government relief programs.

**Results:**

The results show that the average cost of the recommended diet was US$ 1.55 per person/day in Bangkok and US$ 3.76 in Manila (2019 prices in purchasing power parities) immediately before the pandemic. This diet is generally affordable for all households in Bangkok, but only for 37% of households (4.98 million people) in Manila, indicating much higher poverty in the latter. The pandemic and associated government measures decreased the cost of the recommended diet with 6.5% in Bangkok (*p* = 0.001) but not in Manila (*p* = 0.167). Assuming contractions in people’s food budgets of 15–20%, the recommended diet became unaffordable for 0.08–0.12 million people in Bangkok and 6.32–7.73 million people in Manila during the pandemic. Government relief largely compensated for this loss in Bangkok, but relief payments in Manila were not enough to compensate the effect.

**Conclusion:**

These results show that the main effect of the COVID-19 pandemic on the affordability of healthy diets was through the effect on reduced incomes of the poor rather than through prices. Government relief measures should target low-income households to give them the means to purchase healthy food items.

## Introduction

The COVID-19 pandemic severely affected global food security [1]. Even before the pandemic, 690 million people were undernourished, 2 billion people lacked access to safe and nutritious food, and 3 billion were unable to afford a healthy diet [2]. The intake of fruit, vegetables and other healthy foods is of particular concern since these are relatively expensive and were already widely underconsumed before the pandemic [3, 4].

The pandemic threatened food security from two directions: on the supply side, there was reduced farm production as a result of the reduced availability of labor and inputs and the disruption of markets and trade [5, 6]; on the demand side, there was reduced market demand as people lose jobs and income and shift food consumption patterns [7, 8]. While COVID-19 did not discriminate between rich and poor, marginalized people had much fewer options to adapt or cope with income losses, as has been seen in other major health and social shocks [9].

Studies have quantified the global impact of COVID-19 using modeling with assumptions about the shift in income distributions to estimate the number of people who can no longer afford a healthy diet and resulting changes in food consumption [10–12]. Using data from 63 lower- and middle-income countries representing 3.5 billion people, Laborde et al. [11] estimated that 2.5 billion people (70%) could not afford a healthy diet pre-COVID-19, and an extra 141 million people (+4%) could no longer afford it in 2020. Bai et al. [13] compared monthly retail prices for 180 countries from 2019 to 2021 and observed a significant increase in the price of micronutrient-rich food. While these global studies are important to understand the magnitude of the impact, there is also a need for country-specific studies to understand the effects in a local context. For instance, Kang et al. [14] used cross-sectional survey for Bangladesh, India, Indonesia, Myanmar, the Philippines and Vietnam to assess people’s perceptions of food expenditures, availability and affordability while accounting for differences between rural and urban residents. Picchioni et al. [15] highlighted the need for further empirical research evaluating the impact of COVID-19 on the affordability of food.

Building on these studies, we estimate the impact of COVID-19 and its associated mobility restrictions and mitigation policies on the cost of a healthy diet among households in urban areas of Bangkok, Thailand and Manila, the Philippines, using official food price data. Further, we build scenarios using national household food expenditure data to explore the likely effects on the affordability of these diets. Three research questions are addressed: i) How does food expenditure compare to the cost of the recommended diet? ii) What is the impact of the COVID-19 pandemic on the cost of the recommended diet? iii) How has COVID-19 pandemic affected the affordability of the recommended diet?

Urban populations are particularly vulnerable to COVID-19 disruptions as they rely on purchased food and work in industries affected by COVID-19 restrictions such as construction, services, and tourism. Thailand and the Philippines had very different caseloads of COVID-19 by the end of December 2020, and different policy responses making them interesting cases for analysis. Tracking changes in the affordability of foods contributing to nutritious diets in different contexts, and implications for marginalized populations vulnerable to unhealthy diets, is a vital step to informing an evidence-based policy response.

## Material and methods

### Study area and background

We selected Thailand and the Philippines as countries with different early COVID-19 infections, and different sets of policies to mitigate the pandemic’s effects. Our focus on urban and peri-urban areas of Manila and Bangkok, the capital cities, was motivated by the large numbers of infections, marginalized households, and availability of data. The Metro Manila area comprises 16 cities and 1 municipality while the greater Bangkok area consists of Bangkok and its five adjacent provinces. Metro Manila, hereafter simply referred to as Manila, had an estimated population of 13.5 million as of 2020 [16] while the Bangkok Metropolitan region, hereafter referred to as Bangkok, had an estimated population of 16.2 million. Both are the commercial, industrial and political centers of their countries.

On 13 January 2020, Thailand reported its first case of COVID-19, which was also the first case outside China. By 17 March, the country had about a hundred cases per day and the government reacted by imposing lockdown restrictions, including a night-time curfew, cancellations of public events and gatherings, closures of shopping malls, schools and non-essential businesses, work from home orders, and restaurants could only sell take-away food. Strict restrictions on international travel were introduced in the first week of April. By May, near-zero local daily infections were recorded leading to opening of schools and non-essential businesses. A second wave started in December 2020 and by 31 December there were 179 daily confirmed cases, or 2.6 daily new confirmed cases per one million people, and a cumulative number of 7,163 infections [17].

While Thailand managed to cut down COVID-19 cases to nearly zero in 2020, the Philippines was hit hard by the pandemic and it had one of the most stringent and prolonged lockdowns in the world [18]. The country recorded its first case of COVID-19 on 20 January 2020. On 16 March, Manila was put under “enhanced community quarantine”: school activities were suspended, mass gatherings prohibited and working from home encouraged. By the end of May 2020, lockdown restrictions were relaxed with the re-opening of mass transportation, government offices and resumption of work in some sectors. Amid the gradual easing of quarantine restrictions, the Philippines saw an accelerating increase of COVID-19 cases and in August 2020 the government put Manila under modified enhanced community quarantine which lasted for 2 months. Moderate lockdown measures continued into December 2020, by which time the Philippines clocked 1,191 daily confirmed cases, or 10.7 daily new confirmed cases per one million people, and 474,064 cumulative cases [17].

The economic disruptions of the pandemic were felt in both countries. The Thai economy shrank by 6.5% in 2020, household consumption by 1.3 percent, and hours worked by 5.7%; while the official unemployment rate rose from 1.0 percent in the first quarter of 2020 to 2.0 percent in the second quarter [19]. Incomes declined, particularly for low-income households including factory workers, domestic and migrant workers, day laborers, motorcycle and taxi drivers, and street vendors, who mainly live in urban areas. One report estimated that at least half a million migrant workers in Thailand became unemployed due to COVID-19 and that most informal workers lost 70% of their income [20]. Another report showed a 20% drop in income for small business operators [21]. Surveys among informal workers in Thailand estimated income losses of up to 73% [22, 23] or even a loss of all income, especially in Bangkok [24].

The Thai government rolled out various COVID-19 relief and recovery packages in 2020. Measures directly related to income and food spending support included wage subsidies of 62% for formal-sector furlough; 50% of salary but not exceeding the maximum salary of 15,000 baht (US$ 1,156.5 in 2019 prices converted using purchasing power parities - PPP) per month if the employer temporary halts employment [25]; 50% discount on food capped at 3,000 baht (US$ 231.30 in 2019 PPP) per person for 3 months (*Khon La Khrueng* scheme) [26] and cash transfers of 5,000 baht per month (US$ 385.5 in 2019 PPP) for 3 months to informal sector workers (more than half of Thai workers, or 20 million people, are informal workers) and the self-employed, which reached 9 million people in 2020 [27]. Although substantial, this 3-month informal-sector relief is still less than the minimum monthly wage for a single income-earner per household. Besides, marginalized groups such as migrant workers could often not benefit from the same relief programs.

In the Philippines, the economy shrank by 9.5% in 2020, the biggest contraction ever recorded in the country [28]. Unemployment increased threefold in 2020 compared to 2019 reaching 7.3 million individuals, and full-time employment reduced by 50% in the informal sector [29]. A survey of 1,000 residents of Manila showed that 78.1% of poor households were affected by job losses during the lockdown [30]. A World Bank survey of 1,614 low-income households in Manila, estimated an income decline of 36–50% at the beginning of lockdown [31]. A survey by the United Nations Development Programme and the United Nations International Children’s Emergency Fund estimated that 83% of all people in Manila saw their incomes decline, with 40% having lost all income, especially among low-income households [32].

The government of the Philippines introduced new social protection measures, including worker benefits for those affected by furlough or travel bans; unemployment benefits and temporary employment schemes; providing meals or meal packs; and extending, increasing or relaxing of conditionalities on existing benefits. In particular, a government relief program directly linked to income and food support dubbed “Bayanihan to Heal as One Act” provided emergency subsidies for up to 18 million low-income households [33]. Manila residents who qualified for this program could receive two transfers of 8,000 peso (US$ 399 in 2019 PPP), which is just above the Manila minimum wage.

### Cost of recommended diet

Methods for measuring the cost and affordability of a healthy diet have evolved rapidly in recent years. The pioneering work of Herforth et al. [34] proposed using the Cost of a Recommended Diet (CoRD) indicator as applied in a study in Ghana. The CoRD uses the national food based dietary guidelines to estimate the minimum cost. It requires data on food prices and quantitative food based dietary guidelines. CoRD is calculated by identifying the least-cost 2–3 foods, by edible portion, in each food category contained in the food based dietary guideline, and summing the mean cost of obtaining the average gram amounts of each group. Herforth et al. [35] and Mahrt et al. [36] advanced the CoRD method to account for local food preferences within food groups thus creating the cost of recommended diet with food preferences (CoRD-FP) indicator. These studies also introduce two other indicators: the cost of calorie adequacy which measures adequate calories for energy balance using only the least-cost starchy staple; and the cost of nutrient adequacy, which accounts also for other nutrients such as proteins and vitamins [35]. In 2022, the Cost and Affordability of a Healthy Diet (CoAHD) indicator was introduced that deviates from prior measurements, which selected the least cost food items based on price/kg, by selecting least cost items in terms of their price/kcal [37]. We did not use the CoAHD because it was not yet published when we analyzed our data.

We chose the CoRD method for the following reasons: first, the cost of recommended diet uses the food-based dietary guidelines that provide a guarantee for meeting the daily caloric needs in a safe and acceptable manner [38] and was at the time of this research the preferred method; second, CoRD minimizes the bias of including preferences that would lead to a higher cost while just focusing on an energy diet alone is not sufficient for long-term well-being [35].

Estimating the CoRD requires information from FBDGs and the price of each food item, as explained in the following.

#### Food-based dietary guidelines

Developed in 1994, Thai FBDGs are specified for adult workers with different physical activity levels (1600, 2000 and 2400 kcal/day) as shown in Table 1. We are particularly interested in the cost of diet for adults with high physical activity levels as urban poor are more likely to perform heavy manual work. Thailand’s FBDGs include six food groups, namely cereal and cereal products; meat and meat products; vegetables; fruit; milk; and fat, sugar and salt. Nuts, seeds, and pulses are grouped under meat and meat products (protein foods) while herbs and spices such as chili, coriander, and parsley are included under vegetables.

**Table 1:** Recommended diet for adult workers in Thailand for three alternative physical activity levels

Food group	Serving unit	Amount per serving (g)	Servings per physical activity level (kcal/day)		
			1,600	2,000	2,400
Cereals and cereal products	Rice serving spoon	60	8	10	12
Meat, meat products, eggs, seeds, nuts and pulses	Tablespoon	15	6	9	12
Vegetables, herbs and spices	Rice serving spoon	40	4 (6)	5	6
Fruit	Portion	Depends on type and size	3 (4)	4	5
Milk	Cup	200	2 (1)	1	1
Fat, sugar, and salt	Teaspoon (fat & oil)	4–5	5	7	9
	Teaspoon (sugar)	4–5	4	6	8

Source: Sirichakwal et al. [39]

1,600 kcal is advised for children, sedentary women, and elderly; 2,000 kcal is advised for teenagers, young adults, and office working men; 2,400 kcal is advised for those who need more energy such as laborers, farmers, athletes. Portion numbers in parenthesis are recommended for adults. For fruit, we use 120 g per serving which is almost similar to 100 g per serving recommended by Dizon et al. [40] and 100 g suggested by Mahrt et al. [36]

The Philippine FBDGs were developed by the Food and Nutrition Research Institute of the Department of Science Technology in 1990 and were revised to cover different age ranges and populations such as lactating women, pregnant women, elderly people aged 60–69 years, adolescents aged 13–19 years, children aged 7–12 years, and toddlers aged 1–6 years. The guidelines specify the recommended servings for eight food groups: starchy staples, vegetables, fruit, eggs, meat and pulses, milk and milk products, fats and oils, and sugars and sweets. Nuts, seeds, and pulses are grouped under meat. The Philippine FBDGs do not specify quantities for each food group, which is necessary to estimate the CoRD. Yet, Dizon et al. [41] deduced quantities for each food group for people aged 20–39 years and we used these (Table 2). For simplicity, we will refer to cereals and starchy foods as “staples” and meat, fish, and legumes as “protein foods” in both sets of national FBDGs.

**Table 2:** Recommended diet for the Philippines, persons aged 20–39 years

Food group	Recommended servings			Serving size
	Lower bound	Upper bound	Average of bounds	
Rice, rice products, corn, root crops, bread, noodles	5	8	6.5	
– Rice, wheat, flour, noodles				50 g
– Roots and tubers				100 g
Vegetables	3	3	3	100 g
Fruits	2	3	2.5	100 g
Eggs	1	1	1	50 g
Fish, shellfish, meat, poultry, dried beans, nuts	3	4	3.5	
– Fish, meat, poultry				50 g
– Dried beans, nuts				30 g
Milk, milk products	1	1	1	
– Whole milk				240 ml
– Evaporated/condensed milk				120 ml
– Cheese				50 g
– Powdered milk				20 g
Fats & oils	6	8	7	5 g
Sugar & sweets	5	8	6.5	
– Sugar, honey				5 g
– Jams, concentrated juice				10 g

Source: Dizon et al. [41]

*g* Gram, *ml* Milliliter

We follow Raghunathan et al. [42] in calculating the CoRD in six steps: (1) Classify each food item in the price dataset into one of the food group categories identified in FBDGs. (2) Standardize all units to kilograms. For non-standard units (e.g., eggs, bunches) we used conversion factors mainly from Food and Agricultural Organization and the Thai Agricultural Standards [43, 44]. For Thailand, the quantity of staples and vegetables is presented in cooked form and therefore conversion factors are applied, which are from the International Network of Food Data Systems (INFOODS) [45]. Vegetables usually eaten raw (lettuce, tomato, cucumber) were not converted.

(3) Convert all food prices into price per edible serving using the formula:

1 $$\mathrm{Price\, per\, edible\, serving}=\frac{\mathrm{serving\, size\, in\, grams}}{\mathrm{price\, unit\, of\, food\, item\, in\, grams}} /\mathrm{edible\, portion}$$

(4) Select the two items in each food group with the lowest price per serving, and calculate the average price per serving for the food group. (5) Multiply the average price per serving of each food group by the recommended number of servings per food group to obtain the cost of that food group per person per day. (6) The CoRD is the sum of the cost of all food groups. Local prices were converted to 2019 US dollars (US$) using real purchasing power parities (PPP) as derived from the International Comparison Program of the World Bank.^1^ All prices were deflated to 2019 price levels using the consumer price index [46].

#### Regression discontinuity

Regression discontinuity has been used widely to assess the effect of policy changes, including the impact of COVID-19-related measures (e.g. [47–50]). Here, the outcome variable is the CoRD and the treatment is the time when COVID-19 restrictions were imposed, denoted by the dummy variable *Lockdown*. Variable *t* represents monthly time intervals from 2011 to 2020. The time when the first lockdown measures were introduced is denoted as *c*. We hypothesize that the lockdown month represents a significant countrywide economic shock affecting the food retail sector. It follows that COVID-19 Lockdown (*CLockdown)* takes a value of zero in all periods before the start of lockdown and takes the value of one afterward:

2 $${CLockdown}_t=1\;if\;t\geq c\;and\;{CLockdown}_t=0\;if\;t<c$$

The full model is specified as:

3 $${Price}_{i,t}=\alpha + {\beta }_{1}CLockdown+\gamma \left({X}_{i}-c \right)+\mu *{Dummy}_{month}+ {e}_{it}$$

where ${Price}_{i,t}$ is the food group price (or CoRD) *i* at month *t*. ${X}_{i}$ is the assignment variable, $c$ is the value of the cut-off and $\mu *{Dummy}_{month}$ is a vector of dummy variables to control for monthly fixed effects. We include monthly effects to control for seasonal variations in food prices. Our coefficient of interest, ${\beta }_{1}$, estimates the causal effects of the nationwide lockdown on the food price. We employed the default local linear regression discontinuity estimator, triangular kernel, and the Mean Squared Error (MSE) optimal bandwidth [51].

Given that our focus is on the COVID-19 (lockdown) impact, we must identify the cut-off point when restrictions were imposed. Oxford University tracked and compared policy responses to COVID-19 across the world using 17 indicators of government responses expressed as a ‘stringency index’ (0–100) with measures closer to 100 indicating stricter lockdown measures [18]. The average stringency index during 2020 was 49 for Thailand and 70 for the Philippines. The first measures in Thailand were introduced on 5 March 2020 (stringency index = 17) and the highest stringency index of 64 was reported in December 2020. In the Philippines, the first measures were introduced on 23 January 2020 (stringency index = 11) and the highest stringency index was 100 (March and April 2020). Based on the rapid increase in the stringency index, we chose February 2020 as the cut-off month for the Philippines and March 2020 for Thailand.

### Estimating affordability

Some previous studies have assumed that the recommended diet is affordable if its cost is less than 63% of a household’s income (for lower-income countries) [2]. However, the concept of affordability is a subjective one and depends on an individual’s context. In this study, we measure affordability by comparing the predicted mean of the minimum cost of the recommended diet against the mean per capita spending on food: when the cost exceeds actual food expenditures then the diet is considered unaffordable. The cost of recommended diet per person per day and per capita spending are expressed in 2019 US$ PPP.

Food expenditure data for Bangkok and Manila covered the amount of money spent purchasing food and the monetary value of own-produced food and of food received from others. The data included cereals, eggs, milk and dairy products, meat, fish, vegetables, fruit, oil, nuts, and spices. We aligned these categories with those used to calculate the CoRD by including meat, nuts and fish as protein foods, and herbs as vegetables. We included other food expenditures that do not align with the food groups as these also reflect a household’s available food budget. For instance, food eaten outside the home (including non-alcoholic beverages) and prepared food eaten at home (i.e., cooked food bought from outside).

Most previous studies evaluated the effect of COVID-19 using simulated changes in income. For instance, in their study among Filipinos, Albert et al. [52] and Sumner et al. [12] assumed households experienced a 5–20% reduction in income due to the pandemic. However, our background section suggests that these assumptions may be too optimistic. Survey-based reports indicated that marginalized workers in Bangkok and Manila may have experienced a drop in income of 20–70% during the pandemic. Households will smoothen the effect on food consumption as food is an essential commodity. We therefore assumed reductions in per capita food expenditures of 10, 15 and 20% following COVID-19 lockdowns and mobility restrictions. These estimates are similar to actual cuts on food spending among low income households observed in other contexts like America [53].

We also considered the effect of government relief programs offered during the pandemic. We estimated the amount of income support to be US$ 1156.50 per person in Bangkok in 2019 PPP and US$ 414 per household in Manila in 2020. Household expenditure data showed that on average 21% of income is allocated to food in Bangkok and 30% in Manila. We also included the food subsidy provided to those affected in Bangkok which amounted to US$ 231.30 per person. The value of the government relief fund was divided by the number of months under lockdown in the study period. Hence, the food expenditure during the pandemic was the pre-pandemic expenditure contracted by 10, 15 or 20%; plus, the COVID-19-related government relief fund.

### Data used

Retail food prices over a period of 10 years (2011–2020) were obtained from the Department of Internal Trade for Bangkok [54] and from the Philippines Statistics Authority for Manila [55]. Appendices 1 and 2 show the food items obtained from the data and how these were mapped to FBDGs.

The Bangkok data had prices for 225 food items of which 32 were excluded due to gaps in the data (Table 3). The Manila data had prices for 58 food items of which 7 had less than 10 observations and were deleted. Price data for Bangkok covered five FBDG food groups, but not dairy. For Manila, food prices covered five FBDG food groups, but not oil and dairy. The category of sweets and sugars was excluded because prices were unavailable. We obtained the most recent Household Income and Expenditure Surveys (HIES), 2019 for Thailand and 2018 for the Philippines [55, 56] as summarized in Table 4.

**Table 3:** Number of food items and monthly price observations, Bangkok and Manila, 2011–2020

Food group	Bangkok		Manila	
	Food items in the data	Monthly price observations	Food items in the data	Monthly price observations
Staples	12	1,440	8	960
Protein foods	62	7,440	19	2,280
Vegetables	72	8,640	15	1,800
Fruit	31	3,720	8	960
Oils	15	1,800	-	-
Eggs	-	-	1	120
Dairy	-	-	-	-

Sources: Estimated using price data obtained from DoIT [54] and PSA [55]

- represents missing data

**Table 4:** Population characteristics for Bangkok and Manila

Statistic	Bangkok	Manila
Total population (million)	16.2	13.5
Sample size HIES (households)	2,581	17,883
Mean household size (persons)	2.5	4.4
Mean pre-COVID income (US$ in 2019 PPP/person/day)	44	16.53
Government relief during pandemic (US$ in 2019 PPP/household/month)	1387.7	414

Sources: PSA [55], NSO [56]

*HIES* Household Income and Expenditure Survey

## Results

### Cost of recommended diet

We examined foods within each food group with the minimum cost from 2011–2020. The two lowest-cost items in each food group are listed in Appendix 3 which represent the CoRD food basket. Rice was the cheapest staple in Bangkok while rice and corn were the main cheapest staples in Manila. In both cities, seafood and chicken were the cheapest protein food. The cheapest vegetables in Bangkok generally included cauliflower, baby corn, and green gourd while in Manila these included sweet potato tops and cabbage.

The recommended diet cost an average of US$ 1.55 in 2019 PPP (20.1 baht) per person per day in Bangkok (Table 5). Of this, US$ 0.64 (8.3 baht; 41.2%) is the cost of staples, US$ 0.38 (4.9 baht; 24.5%) of fruit, and US$ 0.22 (2.85 baht; 14.2%) of vegetables. At US$ 3.76 in 2019 PPP (75.39 peso) per person per day, the average cost of the recommended diet was twice as high in Manila. Of this, US$ 1.44 (28.87 peso; 38.3%) was the cost of staples, US$ 0.48 (9.64 peso; 12.9%) of fruit and US$ 0.69 (13.83 peso; 18.2%) of vegetables.

**Table 5:** Average CoRD for Bangkok and Manila, before and during COVID-19 pandemic; n = 120; 2011–2020

**Food group**	**Bangkok**			**Manila**		
	Required g per person per day	Cost in US$	Proportion of cost to overall CoRD	Required g per person per day	Cost in US$	Proportion of cost to overall CoRD
Cost of recommended diet (US$ in 2019 PPP/person/day)	-	1.55	-	-	3.76	-
Cost per food group per day:						
– Staples	720	0.64	0.41	325–650	1.44	0.38
– Protein foods	180	0.16	0.10	105–175	0.33	0.09
– Vegetables	240	0.22	0.14	300	0.69	0.18
– Fruit	600	0.38	0.25	250	0.48	0.12
– Oils	36	0.14	0.09	-	-	-
– Eggs	-	-	-	50	0.33	0.09

Sources: Estimated using the price data obtained from DoIT [54] and PSA [55]. Cost in US$ at 2019 PPP

The required g per person per day is the product of the amount of serving and number of servings per person per day. For Thailand, we use number of servings of recommended for those who need more energy such as laborers while for the Philippines we use the recommended servings for average bounds

### The impact of COVID-19 on food prices

The results of the regression discontinuity analysis (Table 6 and Appendix 5 for graphical representation) show that the lockdown led to a rise in the price of vegetables in Bangkok by 0.02 (9.1% increase; *p* = 0.003) but the cost of fruit decreased by US$ 0.08 (21.1% decrease; *p* < 0.011), while the overall cost of the recommended diet declined by US$ 0.1 (6.5% decrease; *p* =  < 0.001). In Manila, COVID-19 lockdown resulted in an increase in the cost of vegetables and fruit of US$ 0.04 (5.7% increase; *p* = 0.001 for vegetables and 8.3% increase; *p* = 0.011 for fruits) and US$ 0.09 for eggs (27.3% increase; *p* = 0.020).

**Table 6:** Regression discontinuity estimates of the impact of COVID-19 on food prices and the CoRD

**Bangkok (*n* = 120)**	**Staples**	**Protein foods**	**Vegetables**	**Fruit**	**Oils**	**CoRD**
Cost per food group (US$/person/day)	-0.228 (0.058)	-0.008 (0.002)	0.016 (0.003)	-0.080 (0.032)	0.021 (0.001)	-0.100 (0.064)
*P*-value	< 0.000	< 0.001	0.013	0.011	< 0.001	< 0.001
Bandwidth estimator right	9	9	9	9	9	9
Bandwidth estimator left	8.2	20	11.6	10.4	10.1	9.5
**Manila (*n* = 120)**	**Staples**	**Protein foods**	**Vegetables**	**Fruit**	**Eggs**	**CoRD**
Cost per food group (US$/person/day)	-0.232 (0.075)	0.018 (0.007)	0.043 (0.013)	0.042 (0.017)	0.091 (0.040)	-0.124 (0.090)
*P*-value	< 0.001	0.013	0.001	0.011	0.02	0.167
Bandwidth estimator right	10	10	10	10	10	10
Bandwidth estimator left	8.5	17.2	20.9	15.5	22	19

Sources: DoIT [54] and PSA [55]

Robust standard errors in parentheses. Under the different specifications, the best bandwidth is calculated using an MSE-optimal bandwidth selector for the RD treatment effect estimator (MSERD)

### Food expenditure and affordability of the recommended diet

Before the COVID-19 pandemic, Bangkok households spent on average 20% and Manila households spent on average 46% of their income on food. In Bangkok, the average person spent US$ 8.98 (2019 PPP) on food daily, of which US$ 6.14 (68%) went to food eaten at home and US$ 2.84 (32%) went to food eaten away from home, while in Manila, the average person spent US$ 7.65, of which US$ 6.13 (80%) was spent on food eaten at home and US$ 1.52 (20%) was spent on food eaten away from home (Table 7).

**Table 7:** Food expenditure and the CoRD before the COVID-19, Bangkok (2019) and Manila (2018)

Food item	Bangkok (*n* = 2,581)				Manila (*n* = 17,883)			
	Food expenditure (US$/ person)	CoRD (US$)	Adequacy	Proportion of income	Food expenditure (US$/ person)	CoRD (US$)	Adequacy	Proportion of income
Food groups:								
– Staples	0.42	0.66	0.64	0.01	0.89	1.44	0.62	0.05
– Protein foods	1.03	0.16	6.44	0.02	1.15	0.34	3.38	0.07
– Vegetables	0.51	0.22	2.32	0.01	0.23	0.69	0.33	0.01
– Fruit	0.7	0.37	1.89	0.02	0.16	0.48	0.33	0.01
– Eggs	-	-	-	-	0.10	0.87	0.11	0.01
– Oils	0.06	0.14	0.43	0.00	0.07	-	-	0.00
Sub-total	2.72	1.56	1.74	0.06	2.60	3.82	0.68	0.16
Other foods:				0.00				0.00
– Milk	0.23	-	-	0.01	0.21	-	-	0.01
– Prepared food	3.19	-	-	0.07	3.32	-	-	0.20
– Food eaten away from home	2.84	-	-	0.06	1.52	-	-	0.09
Total	8.98	-	-	0.20	7.65	-	-	0.46

Sources: PSA [55], NSO [56]

The CoRD is given as per person per day; Adequacy refers to the adequacy of per capita food expenditure compared to the minimum cost of the recommended diet; prop. Of per capita income is the proportion of income spent on food; estimation of prepared food and food away from home includes non-alcoholic beverages

Table 7 also shows the extent to which food expenditures were adequate to meet the minimum cost of the recommended diet: Bangkokians spent on average 1.7 times the minimum cost of the recommended diet while Manila residents spent only about two thirds of what is minimally required to eat healthy. In particular, Bangkokians spent 6.4 times more on protein food than minimally required and this was 2.3 times for vegetables and 1.9 times for fruit; while in Manila, even though the food budget is less than required for a healthy diet, people still spent 3.4 times more on protein food than what is minimally required.

**Fig. 1:**
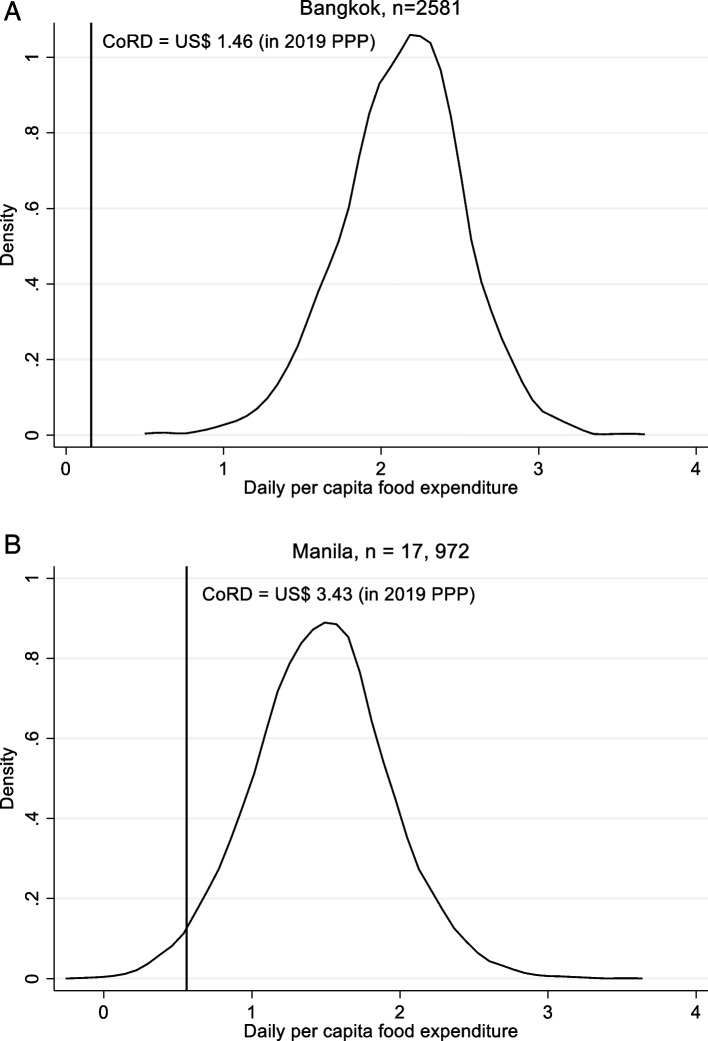
Kernel distribution of daily per capita food expenditure in Bangkok (**a**) and Manila (**b**). *Sources*: Estimated based on PSA [55], NSO [56]; We use predicted CoRD as derived from the postestimation of regression discontinuity

We estimated the proportion of the population that could not meet the minimum cost of the recommended diet (Fig. 1 and Table 8). All households in the data for Bangkok could, in principle, meet the minimum cost of the recommended diet before the COVID-19 pandemic, but 37% of the sample in Manila could not, amounting to 4.98 million people when extrapolated to the whole of Manila (Table 8). During COVID-19 and with a 15–20% contraction of food expenditure, the minimum cost could not be met by 0.08–0.12% of the sample in Bangkok (0.01–0.02 million people if extrapolated to the whole population). In Manila, a 15–20% contraction in food budgets led to a 15–20% increase in the number of people unable to meet the minimum cost of the recommended diet as compared to the pre-COVID-19 situation. This translated into 2.05–2.75 million additional people unable to meet the minimum cost of the recommended diet during the pandemic.

**Table 8:** Population unable to meet the CoRD before and during COVID-19, with and without government relief payments

	Bangkok (*n* = 2,581)				Manila (*n* = 17,883)			
	Without relief		With relief		Without relief		With relief	
	Million people	Proportion	Million people	Proportion	Million people	Proportion	Million people	Proportion
Before	< 0.01	< 0.01	-	-	4.98	0.37	-	-
During:								
–10% food budget	< 0.01	< 0.01	< 0.01	< 0.01	6.32	0.47	5.81	0.43
–15% food budget	0.01	0.08	< 0.01	< 0.01	7.02	0.52	6.46	0.48
–20% food budget	0.02	0.12	< 0.01	< 0.01	7.73	0.57	7.19	0.53

Sources: PSA [55], NSO [56]

We use predicted CoRD as derived from the postestimation of regression discontinuity. The available recent data sets before pandemic are used, i.e., 2018 food expenditure data for Manila and 2019 food expenditure data for Bangkok

However, with the government relief fund provided during COVID-19 pandemic, the minimum cost of the recommended diet would, in principle, be affordable to all persons in Bangkok, assuming that the relief funds were spent evenly over the year. In Manila, the population unable to afford the minimum cost of the recommended diet during COVID-19 would reduce by 3.8% (0.51 million) compared to a situation without relief funds and assuming a 10% contraction of food expenditure. Assuming a 20% contraction of food expenditures, this would reduce by 4% (0.53 million).

## Discussion

We have analyzed the immediate impact of the COVID-19 pandemic and associated policies on the cost and affordability of the recommended diet in Bangkok and Manila. This is one of the first studies to look explicitly at the impacts on food price and affordability, and the first in Asia to use national price, income and expenditure datasets to do this. We find that Bangkok populations pre-COVID were better able to meet the minimum cost of the recommended diet than those in Manila; that COVID-19-related food expenditure contractions have likely pushed millions more people into unaffordability in Bangkok and Manila; and that government relief programs have helped mitigate affordability issues. Methodologically, we extended the CoRD method by applying context-specific food expenditure cut-offs for determining affordability.

### Implications regarding the CoRD

Previous crisis in Asia witnessed a rise in food prices. For instance, the 2008 food price crisis led to a 50% rise in staple food prices and a 21% increase in total food expenditure for low- and middle-income countries, putting pressure on household food baskets [57]. Often the price of the most nutritious foods increases most: In Indonesia, for instance, a drought and financial shock in 1997–8 led to a 200% increase in the price of leafy greens alongside smaller rises in other food prices [58]. However, a key finding of our study is that the price of food did not change much in real-world terms in either city because of COVID-19 during 2020, though the effect varies across food groups and countries. For instance, in Bangkok, the cost of vegetables increased but that of protein foods and fruits reduced; while in Manila the cost of vegetables, fruits, protein foods and eggs increased. The small but consistent increase in the cost of vegetables in both cities may however threaten even the low consumption that existed before COVID-19, where, as shown in our analyses, the per capita spending on this food group in Manila was 67% less than the cost of the minimum recommended diet in 2018. Nutritious foods are reported to be more highly-priced in lower-income countries than in higher-income countries thus limiting the ability of poor households in the former to meet the cost of vegetables and a healthy diet [59], and prices of nutritious foods have been seen to increase during COVID-19 [13, 60] so prices for healthy foods should be monitored routinely, and their price during shocks such as pandemics safeguarded. Disaggregated by city, the minimum cost of meeting the recommended diet is high compared to what an average individual spends on food in Manila, but the cost is lower than what an individual spends on food in Bangkok. Precisely, Bangkok individuals spend 74% more than the minimum cost of the recommended diet; while Manila individuals would need to increase spending by 32%. Breaking down spending by food group, Bangkok people are spending, on average, much more on nutritious foods such as vegetables, protein foods and fruits than the minimum needed to meet the recommended dietary intake of those food groups; while people in Manila are spending much less on vegetables, fruit, eggs and staples. Our findings are consistent with those of Mbuya et al. [61] who, using the 2015 household income and expenditure data, show that households in the Philippines are spending less on vegetables and need to increase spending by 30% to meet the minimum cost of the recommended diet. Similarly, in other parts of the world, like Zambia, urban households are eating less nutritious food because of the COVID-19 pandemic [62].

### Implications regarding affordability

Layoffs and reduced incomes driven by restrictions imposed to combat the virus in the short term are layered on existing food insecurity in many contexts, both in high- and low-income countries. Global economic slowdown and higher poverty rates have been seen in the medium term, and global economic recession is possible in the longer term – all further reducing people’s ability to access nutrient-rich food [63]. The major issue affecting affordability in this study was shocks to food expenditure rather than price; healthy diets based on diverse foods were already too expensive for over 3 billion people in the world before COVID-19 [64]. Despite the cost of the recommended diet in Bangkok reducing during the pandemic, unaffordability under our realistic and context-specific food expenditure contraction estimates has likely increased, especially among poor households in this city, affecting 8–12% (i.e., between 0.01 to 0.02 million) of the individuals. The situation in Manila was worse pre-pandemic, and has likely been exacerbated by COVID-19 with unaffordability increasing from 37% (4.98 million people) to between 47–57% of the population (6.32–7.73 million people). In any given context, the poorest and most vulnerable segments of the population will have fewer resources to cope with loss of jobs and incomes and any increase in the prices of healthy foods, and therefore less ability to adapt to the crisis [63]; in the Association of Southeast Asian Nations 218 million out of the total 649 million population are in informal work [65], and 36 million people live below the poverty line of USD 1.90 per day [66]). A nutrition equity lens [67] is vital in understanding which populations are likely to be most impacted by shocks such as COVID-19 in different contexts; and in planning mitigation measures.

In terms of mitigation, our study demonstrates that the provision of government relief funds improves affordability among poor households in both cities. With this fund, in Bangkok, unaffordability would not be experienced even if households’ food expenditures dropped by 20%; while in Manila each drop of 10, 15 or 20% of food expenditure would lead to a drop of the percentage of individuals not affording by about 5% when compared to a situation without these funds. Our findings support those of Lee et al. [68] who indicate that the social security fund helped the poor in Brisbane, Australia, to afford the recommended diet. It should also be noted however that not all nutritionally vulnerable or marginalized population groups are covered by existing funds; and that fund should be calculated in part based on ability to afford the diet recommended by the same governments providing the funds. Our findings imply that existing and adaptable social security funds are key in alleviating barriers to food affordability during a crisis, as has been seen in political economy work around the pandemic also [69].

### Reflection on the methods

In this study, we combined recent innovations in CoRD methodologies from Bai et al. [13], Raghunathan et al. [42], Masters et al. [70] and others, and extend these through applying context-specific food expenditure cut-offs and contractions for modelling affordability. To our knowledge, this is the first study using national price, income and expenditure data to model the context-specific impacts of COVID-19 on food affordability for urban residents.

The CoRD indicator uses two lowest-cost food items from each food group to estimate the cost of acquiring a recommended diet. The items selected as cheapest are reasonable in the context of Thais and Filipinos. For instance, it is common to find people switching from glutinous rice to regular rice and you can find ginger served as a vegetable. While we could create new ad-hoc rules in the selection process to exclude certain items, it would be arbitrary and deviate from the simple mechanism of CoRD compared to for example CoRD-FP. Since the CoRD uses a few least-cost foods, it may not reflect people’s actual eating behavior or taste preferences, and does also not include the cost of condiments, sauces, as well as cooking gas needed to prepare a meal. For instance, in Bangkok and Manila a lot of urban residents rely on purchased food eaten away from home and many condos do not even have kitchen units.

The study’s food price data does not cover all food groups in included in the FBDGs, as the Bangkok data with 225 items (32 excluded) misses dairy, and the Manila data with 58 items (7 excluded) misses oil and dairy. Although dairy is often a relatively high-cost food group, it is less consumed in most countries in Southeast Asia [36]. Annual per capita milk consumption in Thailand and the Philippines estimated at 26 and 22 kg respectively compared to 287 kg in United States [71].

We compare cost and affordability of a recommended diet using the national estimates of food spending. Other studies estimate affordability using food expenditure defined as the proportion of income spent on food and was given as 0.63 in 2020 [2] for low and middle-income countries but was revised to 0.38 for upper-middle income countries and 0.52 for lower income countries in 2022 [72]. We therefore expect our estimates of the proportion of the population not affording a recommended diet to differ with global estimates such as those of [73] and [74]. Moreso, the CoRD followed in this study uses only two least-cost items per food group while the CoAHD uses 1 to 3 items depending on the food group, and separates the protein food group into legume, nuts and seeds group and animal-sourced protein groups.

The national household income and expenditure data for our study are based on a sample of households that is likely to underrepresent certain low-income groups such as foreign migrant laborers, students living in dormitories, and homeless people. We therefore expect that our finding that < 1% of the Bangkok population was unable to afford the CoRD before the COVID-19 pandemic underestimates the actual number of people unable to afford a healthy diet.

### Reflection on differences in the cost of recommended diets

Differences in the cost of recommended diets could arise from differences in quantities, selected least-cost food items or prices of selected least-cost food items. Given the low cost of diet in Bangkok compared to Manila, one would expect this to be a result of low quantities of food groups. But it is not true for our case. The amount per food group required per person per day is higher for all groups in Bangkok compared to Manila except vegetables which is higher by 60 g for people of Manila (Table 5). We explore whether other factors like selected least-cost items and price could have contributed to the differences. Appendix 3 shows the names of least-cost food items and the frequency that each item enters the least-cost diet. For both cities, rice is selected as the cheapest staple but the specific rice types differ. The least-cost food items reported in protein foods, vegetables and fruit groups differ in the two cities. In Bangkok, most least-cost protein foods and fruit are sold in bundles e.g., banana hand while vegetables are in a mix form e.g., cabbage mix. It could be that food items sold in bundle or mixed form are cheaper than those sold in single pieces or pure form (uniform) as reported in Manila. We show the average price per gram of food items that are similar in Bangkok and Manila and that form the least-cost diets in Appendix 4. The prices of least-cost food items per gram are higher in Manila than in Bangkok. Rice price is 2 times higher while vegetable and fruit prices are 3–6 times higher in Manila than in Bangkok. Evidently, the high cost of diet in Manila could be partly due to prices.

## Conclusion

Before the COVID-19 pandemic, a healthy diet was in principle affordable to nearly all people in Bangkok, but only for 37% of the population in Manila. The COVID-19 pandemic slightly reduced the cost of a healthy diet in Bangkok, but increased it in Manila. The main effect of the COVID-19 pandemic on diets was through a reduction of household food budgets as people lost jobs and income sources. The exact magnitude of this contraction is unknown, but assuming a 15–20% drop in food budgets would mean that healthy diets became unaffordable for 0.01–0.02 million people in Bangkok and 6.31–7.73 million people in Manila. Our analysis shows that government relief measures were important to maintain people’s food expenditures. Special care needs to be taken to target relief measures to the poor, as they lack other social safety nets to compensate income losses. Therefore, policy-makers are faced with the future issue on vulnerability to poverty due to the shocks to food expenditure which require attention and action.

## Figures and Tables

**Fig. 2:**
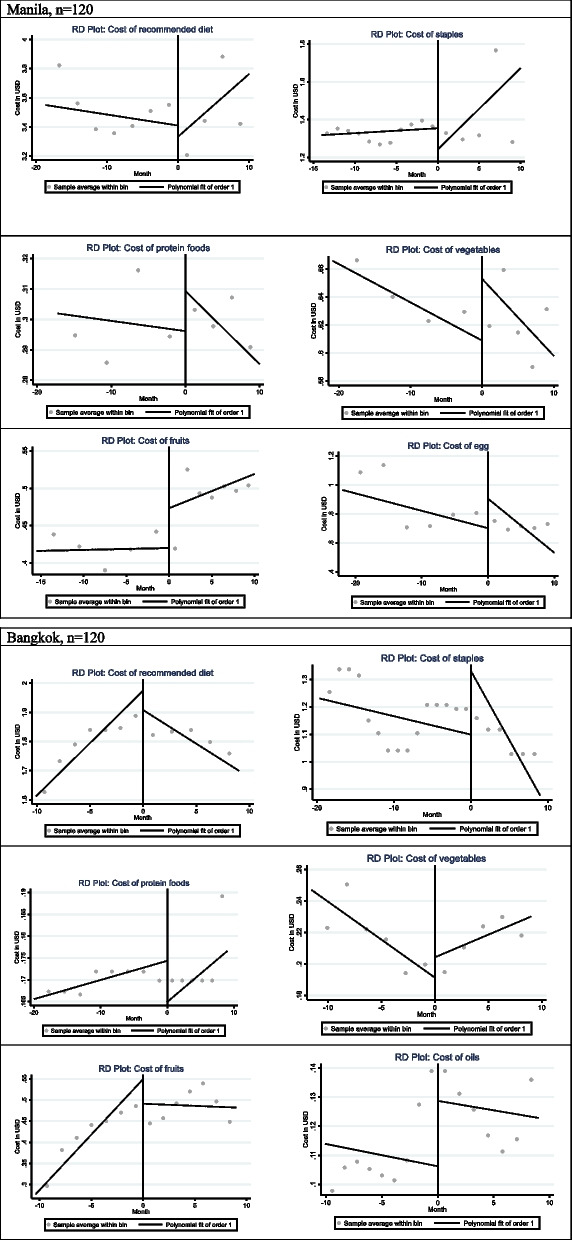
Regression discontinuity plots

## Data Availability

Data used for this study are available from the National Statistics Office in Thailand and the Philippine Statistics Authority and can be requested from these organizations.

## Appendix 1

**Table 9:** List of food items from Bangkok dataset, classified based on Thailand FBDG

Food group	Food type	List of food items
Staples	Rice	Sticky rice 5%, Sticky rice, Sticky rice short, Sticky rice long, Khao san 5%, general rice, Khao san 5% new rice, Pure rice ordinary, Pure new rice, Fragrant general rice, Fragrant new rice
	Roots	Potatoes grade A, Potatoes grade B, Potatoes grade O
	Others	Millet crackers
Protein foods	Eggs	**Chicken**: Chicken egg number 0, Chicken egg number 1, Chicken egg number 2, Chicken egg number 3, Chicken egg number 4, Chicken egg number 5 **Duck**: Duck egg medium, Duck egg small, Duck egg big, Salted duck egg medium, Salted duck egg big
	Meat	**Chicken**: Chicken legs, Chicken offal, Chicken carcass, Chicken drumstick, Chicken fillet, Chicken breast, Chicken wings, Chicken wingstick, Chicken with skin, Whole chicken without offal, Whole chicken with offal **Buffalo**: Buffalo meat, Buffalo meat striploin, Buffalo meat tenderloin **Beef**: Beef, Beef sirloin, Beef tenderloin, Beef with bone, Boneless beef foreleg, Boneless beef hindleg, Beef shank, Beef streaky **Pork**: Pork hip no cutting, Pork hip, Pork shoulder no cutting, Pork shoulder cutting, Pork loin, Pork tenderloin, Pork streaky, Pork back fat, Pork fat leaf, Pork fat scrap
	Sea food	Thai pond lobster medium, White shrimp 40 pcs, White shrimp 50 pcs, White shrimp 60 pcs, White shrimp 70 pcs, White shrimp 80 pcs, Banana shrimp, Shrimp 40 to 50 pcs, Snakehead fish, Catfish, Tilapia, Mackerel 3 pieces, Mackerel 9 to 12 pieces, Tilapia new, Sawai fish, Cuttle fish assorted, Squid assorted, Cockels assorted, Mussels mixed, Clams mixed
	Seeds and nuts	White sesame seeds, Black sesame, Shelled peanuts mixed, Peanuts shelled selected, Special shelled peanuts, Shelled peanut normal selection, Coconut grated
Vegetables		Cauliflower mixed, Cauliflower selected, Cabbage mixed, Cabbage selected, Baby corn, Young ginger, Old ginger, Celery mixed, Celery selected, Spring onion mixed, Ton onion selected, Cucumbers mixed,Cucumbers selected, Yard long beans mixed, Yard long beans selected, Cantonese vegetables mixed,Cantonese vegetables selected, Vegetables in shading, Chinese cabbage mixed, White cabbage selected, Lettuce mixed, Lettuce sorted, Kale mixed, Pickled kale, Coriander assorted, Coriander mixed, Water spinach mixed, Water spinach selected, Thai morning glory, Chilli Chinda, Fresh chilli, Capsicum mixed, Capsicum selected, Eggplant, Big Tomatoes mixed, Big tomatoes selected, Sida Tomatoes mixed, Sida tomatoes selected, Chinese bitter gourd mixed, Chinese bitter gourd selected, Asparagus mixed, Asparagus selected, Beets mixed, Beets selected, Dried okra, Dried garlic cloves small, Dried garlic cloves large, Dried garlic head medium, Dried garlic head small, Dried garlic head big, Dried garlic, Dried garlic small head cork, Dried garlic bunch big head, Dried chilli, Second dry chilli, Quality white pepper, Quality black pepper, Shallots cut medium, Shallots cut small, Shallots cut big, Shallots tied medium, Shallots tied small, Shallots tied big, Shallots Sisaket cut medium, Shallots sisaket tied medium, Shallots sisaket tied small, Shallots sisaket tied big, Onion awaiianuri 0–1, Onion awaiianuri 2, Red onion small, Red onion big, Onion san number 0–1, Onion san number 2, Onion cold storage number 0–1, Onion cold storage 2, Onion fang number 0–1, Onion fang 2
Fruits		Golden banana medium, Golden banana big, Cultivated banana, Gros Michel banana big piece, Gros Michel banana big 14 to 15 pcs, Rambutan, Kinnaree watermelon, Durian chani, Durian monthong, awaii, Guava assorted, Guava selected, Green mango number 0, Green mango number 1, Barracuda mango number 0, Barracuda mango number 1, Keakdam papaya, Holland papaya, Mangosteen rough skin, Mangosteen oily skin, Southern langsat, Longan, Emperor lychee, Hon huay lychee, Orange number 4, Orange number 5, Orange number 6, Pomelo white gold small, Pomelo white gold big, White pomelo honey small, White pomelo honey large, Pineapple number 1, Pineapple number 2, Pineapple number 3, White grape assorted, White grape selected, Lemon numbers 1–2, Lemon numbers 3–4, Coconut fruit, Tamarind seed carving, Tamarind without seed
Oil		Pure peanut oil 5 lit, Pure peanut oil 13 lit, Pure peanut oil 1 lit, Pure peanut oil 2 lit, Pure soybean oil 1 lit, Soybean grape oil 1 lit, Sunflower oil, Sunflower grape oil, Sunflower shim oil, Palm oil bag, Palm oil bottled, Coconut oil, Prepackaged coconut oil, Roi coconut oil, Coconut oil tin, Coconut oil lettuce, Coconut oil p, Coconut oil pail, Arosa rice bran oil, King rice bran oil, Chim rice bran oil

Source: Food items obtained from DoIT [54]

## Appendix 2

**Table 10:** List of food items from the Manila dataset, classified based on the Philippines FBDG

Food group	Food types	List of food items
Staples	Rice	Regular milled rice, Rice premium, Rice special, Well-milled rice
	Roots	Cocoyam, Sweet potato, Irish potato
	Corn	Corn grain white, Corn grain yellow, Corn grits white, Corn grits yellow
Protein foods	Meat	Beef lean meat, Beef meat with bones, Pork lean meat, Pork meat with bones, Pork leg, Chicken boiler live, Chicken broiler dressed
	Sea food	Bisugo threadfin_bream, Blue crab, Dalagang bukid, Anchovy, Galunggong, Indian mackerel, Milk fish, Slipmouth, Shrimp (fresh water), Prawn, Tilapia, Tulingan,
	Seeds and nuts	Peanut with shell, Peanut without shell
Vegetables		Cabbage, Water spinach, Pechay, Sweet potato tops, Carrots, Bitter melon, Chayote, Eggplant purple, Squash, Tomato, Upo bottle gourd, Garlic, Ginger awaiian, Onion red creole, Onion white
Fruits		Banana lakatan ripe, Banana latundan ripe, Banana saba ripe, Lemon, Mandarin, Mango ripe, Mango piko ripe, Papaya, Pineapple
Egg		Chicken egg, Duck egg

Source: Food items obtained from PSA [55]

## Appendix 3

**Table 11:** Common foods selected as the lowest cost items for each food group, 2011–2020

**Bangkok**				**Manila**		
**Food group**	**Food item**	**Frequency (** ***n*** ** = 120)**		**Food item**	**Frequency (** ***n*** ** = 120)**	
		Cheapest	Second cheapest		Cheapest	Second cheapest
Staples	Rice khaosan new	120	0	Regular milled rice	43	28
	Rice khaosan general	0	119	Yellow corngrits	3	4
	Rice pure new	0	1	Premium rice	55	14
				Well milled rice	12	45
				Special rice	2	24
				Irish potato	5	0
				Cocoyam	0	5
Protein foods	Mackerel 9 piece	28	92	Threadfin bream fish	111	8
	Mackerel 3 piece	92	26	Tilapia fish	9	71
	Chicken offalls	0	2	Slipmouth fish	0	37
				Tuna fish	0	2
				Dressed chicken	0	1
				Snap beans	0	1
Vegetables	Cauliflower mixed	12	20	Sweet potato leaves	120	0
	Cabbage mixed	6	9	Cabbage	0	109
	Cantonese mixed	3	3	Water spinach	0	4
	Baby corn	28	46	Hawaiian ginger	0	5
	Lettuce	1	4	Bitter gourd	0	1
	Water spinach mixed	4	7	Carrots	0	1
	Green gourd	66	29			
	Kale mix	0	1			
	Beets mixed	0	1			
Fruit	Pineapple 3 piece	40	30	Philippine lemon	70	50
	Golden banana medium	73	24	Mandarin orange	50	50
	Cultivated banana/hand	7	46	Saba banana	0	13
	Watermelon	0	1	Pineapple	0	5
	Pineapple 2 piece	0	11	Mango	0	2
	Golden banana big	0	7			
	Mangosteen	0	1			
Oil	Palm oil bag	115	1		-	-
	Palm oil bottle	5	102		-	-
	Coconut oil pail	0	9		-	-
	Soybean oil	0	3		-	-
	Soybean oil 1 kg	0	4		-	-
Chicken egg					120	

Sources: Food items and prices from the DoIT [54] and PSA [55]

## Appendix 4

**Table 12:** Comparing average price per gram of least-cost food items that are similar in Bangkok and Manila for the period of 2011–2020

**Bangkok**			**Manila**		
**Item**	**Price per g (US$)**	**n**	**Item**	**Price per g (US$)**	**n**
**Staples**					
Rice khaosan new	0.002	120	Regular milled rice	0.004	115
Rice khaosan general	0.002	120	Premium rice	0.004	115
Rice pure new	0.002	120	Well milled rice	0.004	115
		120	Special rice	0.004	115
**Vegetable**					
Cabbage mix	0.001	120	Cabbage	0.005	120
Water spinach mix	0.002	120	Water spinach	0.005	120
**Fruit**					
Pineapple 2 piece	0.002	120	Pineapple	0.006	120
Golden banana medium	0.001	120	Saba banana	0.006	120
Cultivated banana/hand	0.002	120			
Golden banana big	0.001	120			

Sources: Food items and prices from the DoIT [54] and PSA [55]. The US$ is in 2019 PPP

## Abbreviations

CoRD: Cost of a recommended diet
CoRD-FP: Cost of a recommended diet with food preferences
COVID-19: Coronavirus disease 2019
FBDG: Food-Based Dietary Guidelines
PSA: The Philippine Statistics Authority
NSO: National Statistics Office (of Thailand)
USD: United States Dollar

## Footnotes

1 Purchasing power parities released by the International Comparison Program (ICP) are a more direct measure of what money can buy than exchange rates when local prices are compared internationally.
